# New Appliances and Things Medical

**Published:** 1906-11-03

**Authors:** 


### NEW APPLIANCES AND THINGS MEDICAL.

BORAX, BORICATED SOAPS, AND POWDERS.

(The Patent Borax Co., Ltd., 53 King William Street.)

This company have submitted to us samples of "pure antiseptic borax," of "boricated glycerine superfatted soaps," and of a "borax powder" for the cleansing of sponges and brushes. Perhaps few people are aware that borax, as a new trade product, was first introduced to the world by Mr. Arthur Rowbottom, whose name we are glad to see on the packet of borax before us. Equally few credit Mons. Pasteur with the introduction of boric acid into modern surgery, where it is still largely used by men like Sir Victor Horsley for the purpose of wound-cleansing and the prevention of infection, because it can be tolerated in the majority of cases without injury to the skin. It is used as the basis for dusting-powders for hair-washes, and on account of its alkaline reaction, its non-irritating qualities, and its ready solubility in water it contains in itself all the qualities of a mouth-antiseptic. Being soluble in glycerine it forms an excellent glycerine soap very suitable for tender or chapped skin. The specimens before us are excellent in every respect. They contain an excess of fatty material. Civilisation has rendered the skin dry and tender, and it is important for the sake of personal comfort to keep it more or less greased, and this is accomplished in the most hygienic manner by this borax toilet transparent soap and the glycerine superfatted soap prepared by this company. Their products are of a high and uniform standard of purity and excellence.

